# The digital transformation of medical education collaborative governance in the era of smart education: an innovative management restructuring of medical talent cultivation systems

**DOI:** 10.3389/fmed.2026.1781836

**Published:** 2026-04-24

**Authors:** Xiaze Zhang, Pei Shi

**Affiliations:** 1 Department of Teaching Management of First Clinical College, Heping Hospital Affiliated to Changzhi Medical College, Changzhi, Shanxi, China; 2 Department of Internal Medicine of First Clinical College, Heping Hospital Affiliated to Changzhi Medical College, Changzhi, Shanxi, China

**Keywords:** digital governance, intelligent assessment, learning analytics, medical education management, medical-education collaboration, smart education

## Abstract

Smart education, supported by artificial intelligence (AI), big data, and virtual simulation, is reshaping medical education toward more flexible and collaborative models. However, persistent barriers—fragmented governance, limited data interoperability, and uneven resource distribution—continue to constrain effective collaboration between medical schools and teaching hospitals. This narrative review synthesizes recent developments in digital governance for medical education and proposes a four-layer framework comprising institutional foundations, cross-organizational mechanisms, platform-based technological support, and data-driven quality assurance. The review also highlights key implementation considerations, including interoperability standards for integrating educational and clinical data, staged technology adoption across different resource settings, and governance-oriented evaluation criteria for AI-enabled assessment (e.g., effectiveness, reliability, fairness, explainability, and human oversight). Finally, it discusses privacy, security, and algorithmic accountability challenges under different regulatory contexts. Overall, smart education may facilitate a transition toward more data-informed governance, while requiring context-sensitive implementation and robust ethical safeguards.

## 1 Introduction

Medical education is undergoing rapid transformation in response to demographic shifts, changing disease patterns, and increasing public health demands. These changes have made the cultivation of medical professionals more urgent than ever (1). However, many medical education systems continue to face persistent challenges, including outdated curricula, limited access to high-quality clinical training resources, and misalignment between educational processes and ongoing healthcare reforms (2). As a result, both educators and healthcare professionals are re-examining how medical education can more effectively respond to evolving clinical and societal needs.

In recent years, emerging technologies such as artificial intelligence (AI), virtual simulation, and learning analytics have been increasingly integrated into educational practice. These tools are reshaping instructional models and learning modalities (3). Smart education emphasizes learner-centered design and provides adaptive, personalized support through intelligent technologies. This approach closely aligns with the competency-based, practice-oriented, and process-evaluated paradigms in modern medical education (3, 4). Against this backdrop, the technological boundaries of medical education continue to expand, and teaching methodologies and learning trajectories are shifting toward more open, flexible, and individualized formats.

Despite these technological advances, the cultivation of medical professionals in the smart education era is not merely a matter of technological adoption. The traditionally fragmented governance structure—comprising medical schools, teaching hospitals, and regulatory authorities—has led to misalignment across educational objectives, curricular frameworks, clinical training, and quality assessment (5). Data silos, fragmented resources, and limited collaboration further hinder the coherence and systemic integration of medical education, diminishing the transformative potential of smart technologies (6). Addressing these challenges requires reforming traditional governance models and establishing more integrated, efficient collaborative mechanisms across organizations, institutions, and resource domains.

Therefore, this review explores the digital transformation of collaborative governance in medical education from the perspective of smart education, and examines how this transformation reshapes the medical talent training system. This article outlines the literature selection strategy, reviews the conceptual foundations of smart education, and analyzes the current challenges faced by collaborative governance. Subsequently, a digital governance framework is proposed, and key issues related to data governance and intelligent assessment are discussed. Finally, strategic directions for future development are proposed to support the establishment of a more open, collaborative, and intelligent medical education ecosystem and to enhance the overall quality of medical talent cultivation.

## 2 Literature selection strategy

This study was conducted as a narrative review. Relevant literature was identified through searches of PubMed, Web of Science, and Scopus databases using keywords including “smart education,” “medical education,” “digital governance,” and “learning analytics.” Publications published between 2010 and 2025 were primarily considered. Literature screening was guided by predefined inclusion and exclusion criteria. The inclusion criteria comprised peer-reviewed articles published in English that addressed digital transformation in medical education, collaborative governance models, or intelligent assessment systems. The exclusion criteria included studies not directly related to medical education or digital governance, as well as conference abstracts, editorials, and commentaries without substantive analytical content. Articles were then selected based on their relevance to digital transformation in medical education, collaborative governance models, and intelligent assessment systems. The retrieved literature was qualitatively synthesized through thematic analysis to identify key themes and conceptual developments in this field.

## 3 Conceptual and technological foundations of smart education

In both classroom and clinical settings, medical learners are frequently confronted with complex knowledge structures, context-specific tasks, and intensive skill training requirements. Traditional instructional approaches are increasingly insufficient to address these multifaceted challenges. Smart education has emerged as a learner-centered paradigm providing flexible and adaptive support based on individual differences, while also enabling instructors to dynamically adjust teaching strategies (7). It represents not only a technological innovation but also a systemic response to the persistent structural challenges in medical education.

**FIGURE 1:**
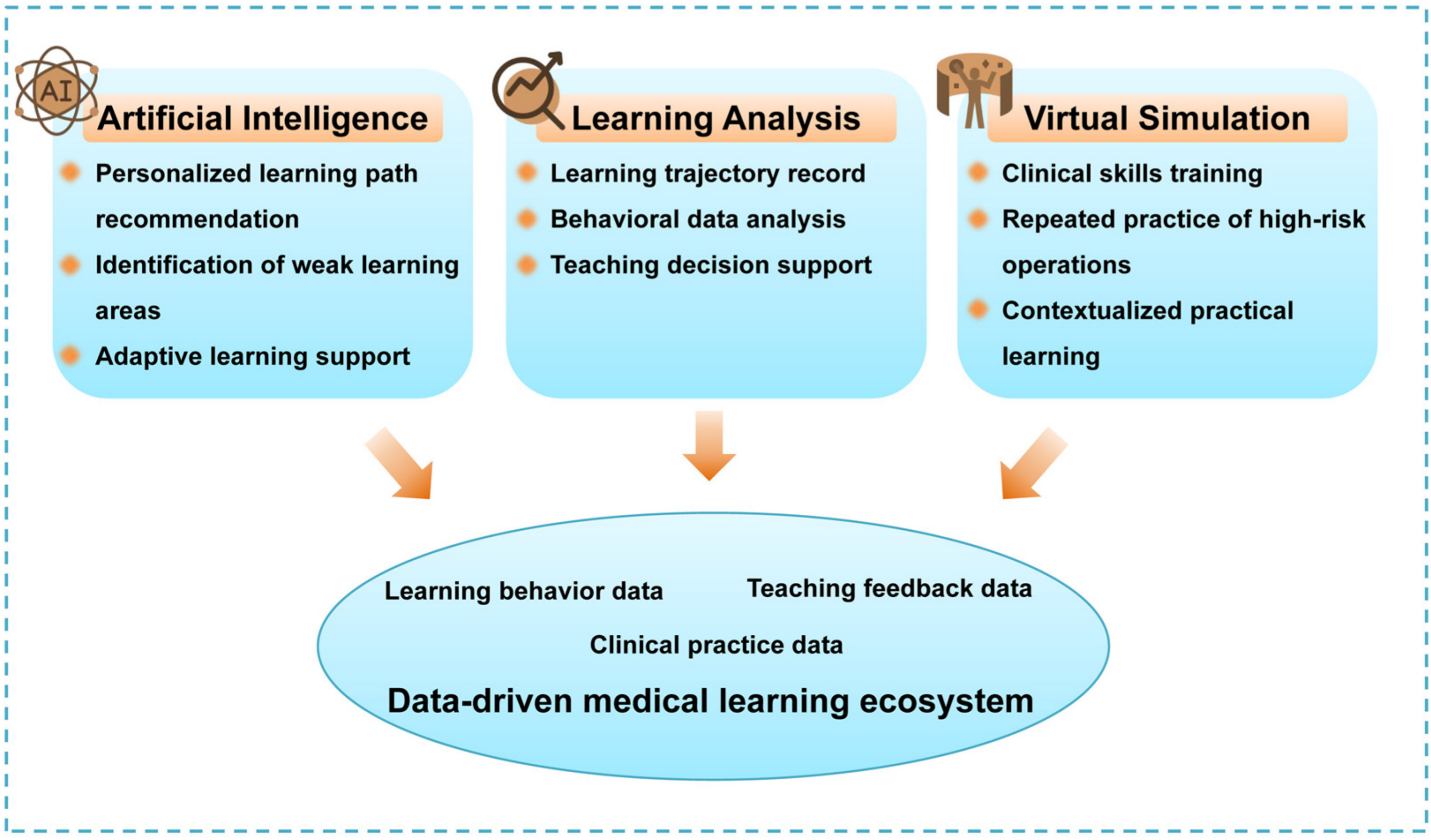
Technological framework supporting smart education in medical training.

Importantly, the principles of smart education have moved beyond theoretical constructs and are now embodied in a range of practical and operational technologies. Virtual simulation platforms allow students to repeatedly practice clinical skills, overcoming physical resource constraints (8). AI assists in identifying individual learning weaknesses and delivering personalized interventions (3). Learning analytics platforms continuously track student learning trajectories, providing evidence-based support for instructional decisions (9). Collectively, these technologies have formed a new data-centric learning ecosystem, as illustrated in Figure 1. Key digital technologies supporting smart medical education and their governance relevance are summarized in Table 1.

**TABLE 1:** Key digital technologies supporting smart medical education.

Technology	Educational application	Governance relevance
Artificial Intelligence	Personalized learning, predictive analytics	Supports adaptive governance
Learning Analytics	Monitoring learning behaviors	Data-driven decision making
Virtual Simulation	Clinical skill training	Enhances training scalability
Medical education data platforms	Integration of teaching and clinical data	Facilitates cross-institutional collaboration
Intelligent assessment systems	Automated competency evaluation	Supports continuous quality monitoring

The advancement of smart education has not occurred overnight but has evolved through ongoing practice and iterative refinement. For example, some instructors have adopted intelligent recommendation systems to provide tiered case-based learning materials tailored to students’ proficiency levels. Simulation-based training centers have leveraged virtual technologies to enhance students’ procedural fluency. In assessment, real-time feedback mechanisms based on behavioral data are increasingly integrated into the learning process (10, 11). These practices indicate that smart education is not merely a collection of tools but a systemic force embedded throughout the teaching process, continuously driving the transformation of medical education.

Therefore, the conceptual and technological foundations of smart education serve as the groundwork for the digital transformation of medical education. Enabled by these principles and tools, medical teaching is no longer confined to physical classrooms or limited by resource availability, but is advancing toward more precise, flexible, and learner-focused models.

## 4 Current status and challenges of collaborative governance between medical schools and teaching hospitals

The medical education system comprises three core actors: medical schools, teaching hospitals, and regulatory authorities. Together, they aim to build a comprehensive talent development pipeline through theoretical instruction, clinical practice, and quality management (12). However, in practice, the level of coordination among these entities remains suboptimal. Medical school curricula often lag behind the evolving needs of clinical services; students experience difficulties transitioning between theoretical learning and clinical application; and clinical instructors, constrained by heavy clinical workloads, are frequently unable to fully engage in teaching activities. These issues reflect longstanding structural tensions in medical-education collaboration.

**FIGURE 2:**
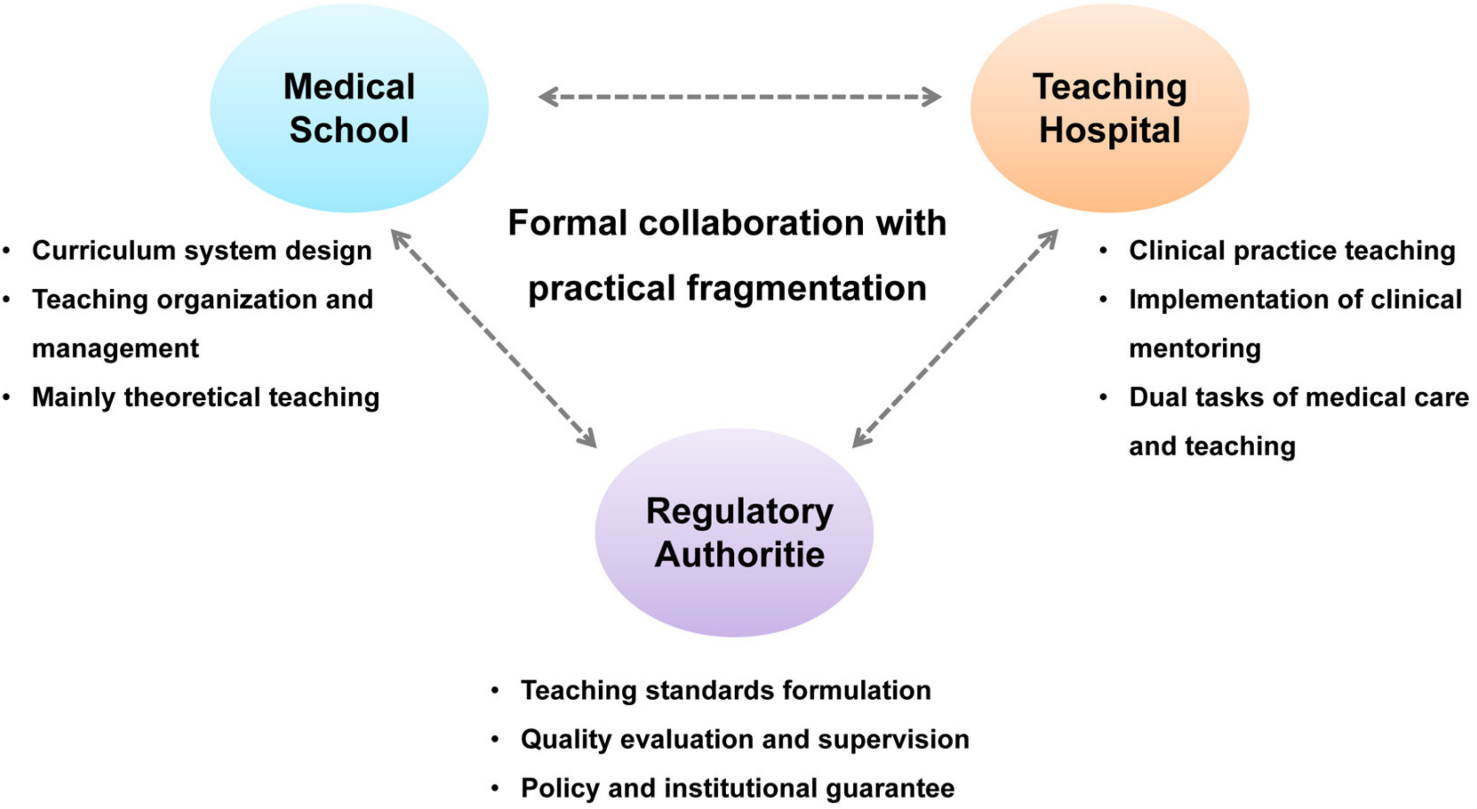
Governance structure of medical-education collaboration.

From a governance perspective, while the responsibilities of each stakeholder are relatively well defined—medical schools manage curriculum design, hospitals oversee clinical training, and regulators establish standards (Figure 2)—there is a lack of systemic integration across processes. Information flow, resource allocation, and decision-making remain weakly connected, resulting in what is often described as “formal collaboration with practical fragmentation.” For instance, curriculum design seldom incorporates timely input from frontline clinical practice, and hospitals’ accumulated clinical data rarely inform instructional resource development or quality assessment.

Data silos and fragmented resources represent major obstacles to the digitalization of medical education systems. Medical schools and teaching hospitals often develop information systems independently, resulting in inconsistent data standards and limited interoperability. Consequently, learning records, clinical training data, and instructional feedback remain difficult to integrate (13). International experiences demonstrate how governance structures and data standards shape digital integration in medical education. In the United States, the decentralized organization of medical education and healthcare institutions often leads to institution-specific information systems, which may limit interoperability and the linkage of educational and clinical data across institutions (14, 15). By contrast, the United Kingdom has established nationally coordinated mechanisms for linking educational and training data. The United Kingdom Medical Education Database (UKMED) integrates undergraduate and postgraduate training data across institutions, enabling longitudinal analyses of training outcomes and workforce development (16). In China, recent reforms have increasingly promoted consortium-based collaboration between medical schools and healthcare institutions. Medical and health consortia have been used to strengthen coordination in clinical training, continuing medical education, and resource sharing, reflecting a hybrid governance approach that combines institutional flexibility with coordinated system development (2, 17). Taken together, these international experiences suggest that the degree of national coordination in governance arrangements and data standards can significantly influence the development of interoperable and longitudinally linked training data systems in medical education. A comparative summary of these governance models is presented in Table 2.

**TABLE 2:** Comparative summary of international governance models for digital medical education systems.

Country/region	Governance structure	Data integration mechanism	Key advantages	Challenges
United States	Decentralized institutional governance	Institution-specific systems	Institutional autonomy and innovation	Limited interoperability
United Kingdom	National coordination via UKMED	Integrated national training data	Longitudinal training data linkage	Data governance complexity
China	Medical education consortia	Regional collaborative platforms	Resource integration and policy coordination	Regional variability

UKMED, United Kingdom Medical Education Database.

Beyond governance structures, limited digital readiness further constrains collaborative governance. Many institutions still lack comprehensive digital management frameworks and clear guidelines for data governance, resource sharing, and privacy protection (18). Differences in faculty digital literacy and disparities in technological infrastructure across hospitals further complicate coordinated digital transformation (19).

Overall, the challenges facing collaborative governance in medical education arise from a combination of fragmented resources, incompatible data systems, and uneven institutional capacities. In the context of rapidly advancing smart education, addressing these structural barriers has become essential for achieving effective digital integration in medical education systems.

## 5 A digital governance framework for medical education

**FIGURE 3:**
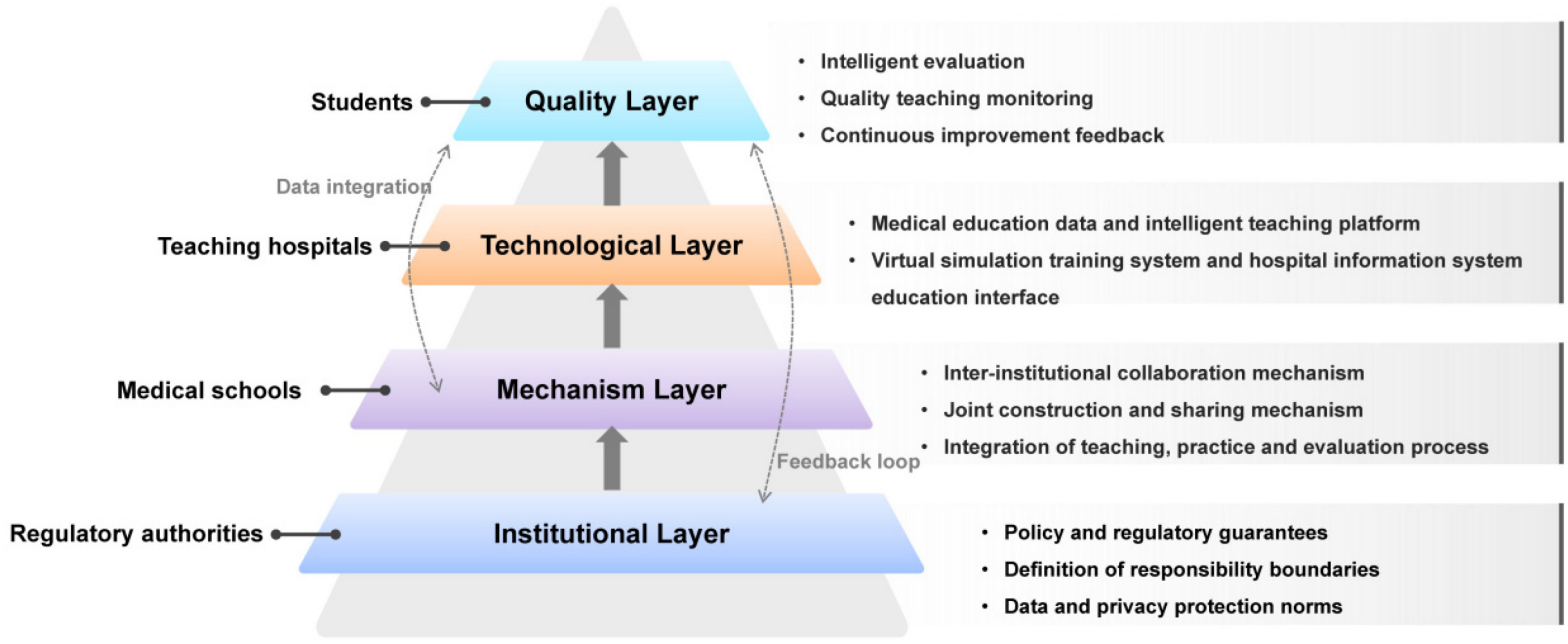
Conceptual framework of digital governance for medical education. The framework comprises four interrelated layers—institutional, mechanism, technological, and quality. Arrows indicate data flow and feedback loops across layers. Key stakeholders (medical schools, teaching hospitals, regulatory authorities, and students) are incorporated to reflect their roles in the system. Together, the framework represents a closed-loop, data-driven governance structure.

To optimize collaborative governance in medical education, a digital restructuring of its governance architecture is imperative. Effective coordination among instructional design, clinical training, and quality assurance requires systemic integration. Building on this need, this section proposes a digital governance framework comprising four interrelated components—institutional, mechanism, technological, and quality layers—aimed at enabling a sustainable and data-driven talent development system through top-level design and platform integration (Figure 3).

Previous studies in education and health systems research have proposed various multi-level governance and systems-based frameworks to analyze institutional coordination and policy implementation (6, 20). These models typically emphasize interactions among policy structures, organizational mechanisms, technological infrastructure, and monitoring systems. The framework proposed in this study builds upon these perspectives but adapts them to the context of digitally enabled medical-education collaboration. By integrating institutional design, cross-organizational coordination mechanisms, digital platform infrastructure, and data-driven quality monitoring, the model highlights the role of interoperable educational data systems and intelligent assessment technologies in supporting coordinated governance and medical talent cultivation (21). However, the implementation of digital governance frameworks may vary across regions and institutions due to differences in technological infrastructure, financial resources, and organizational capacity. Therefore, the framework proposed in this study should be interpreted as a conceptual model intended to guide context-sensitive and phased implementation. In practice, institutions may adopt a staged implementation pathway (22). Initial efforts may focus on establishing interoperable data infrastructures and governance standards. At intermediate stages, learning analytics systems can be integrated to support monitoring and feedback (23). At more advanced stages, AI-driven predictive models and intelligent assessment tools may be incorporated to enable adaptive governance and personalized training management (24).

### 5.1 Institutional layer: top-level design and regulatory foundation

The institutional layer forms the core foundation of digital governance. Its primary function is to establish a stable, compliant basis for collaborative medical-education governance by clarifying policy frameworks, role delineation, and data governance standards. Key stakeholders in this governance framework include medical schools, teaching hospitals, regulatory authorities, faculty members, and medical trainees (25, 26). Each stakeholder contributes to different aspects of governance, including policy formulation, clinical training management, data governance, and educational quality monitoring (27).

First, the responsibilities of medical schools, teaching hospitals, and regulatory bodies should be clearly defined in areas such as instructional organization, resource management, and data usage. This reduces operational fragmentation and avoids unclear accountability. Second, a unified data governance framework should be established, encompassing data collection standards, access control protocols, and privacy protection mechanisms. A sound institutional infrastructure is essential for the secure and sustainable deployment of smart education technologies.

### 5.2 Mechanism layer: organizational and operational systems for cross-institutional collaboration

Building on the institutional foundation, the mechanism layer focuses on the operationalization of collaboration, aiming to bridge organizational boundaries and streamline the flow across teaching, clinical practice, and feedback loops.

Developing co-construction and resource-sharing mechanisms facilitates integrated planning for curriculum development, clinical scheduling, and resource allocation. Standardized data exchange interfaces and unified workflows enable real-time cross-institutional transmission of instructional activities, student performance data, and clinical records (28). This layer improves educational management efficiency and supports the seamless operation of smart education platforms.

### 5.3 Technological layer: systemic and platform-based support for digital governance

The technological layer serves as the infrastructure backbone for digital governance. It consists of core systems such as medical education data platforms, smart teaching platforms, virtual simulation systems, and educational interfaces within hospital information systems.

The medical education data platform consolidates teaching, clinical, and evaluation data to support learning analytics and data-informed decision-making. Smart teaching platforms use AI and analytics to deliver personalized learning pathways and real-time feedback. In practice, AI functions in medical education governance typically fall into three categories: risk prediction (early identification of struggling trainees), decision support (recommendations for learning pathways), and automated or semi-automated assessment (scoring and feedback generation) (29–31). Clarifying the target task and required data inputs is essential, because evaluation criteria and governance risks differ substantially across these use cases. Virtual simulation enhances the immersion and repeatability of clinical skill training. Hospital information systems, when connected through educational interfaces, allow clinical data to inform instructional content and assessment (32–34). The interoperability and coordination among these platforms are vital for effective implementation of digital governance.

Effective interoperability among educational and clinical systems typically relies on standardized data exchange architectures. In healthcare environments, interoperability is often supported by data standards such as Health Level Seven Fast Healthcare Interoperability Resources (HL7-FHIR), which enables structured exchange of clinical information across hospital information systems (35). Within educational technology ecosystems, interoperability protocols such as Learning Tools Interoperability (LTI) and Experience API (xAPI) facilitate the integration of learning platforms and the tracking of learning activities across distributed environments (36, 37). In addition, privacy-preserving approaches such as federated learning may enable collaborative development of AI models across institutions without directly sharing sensitive educational or clinical data (38, 39). These interoperability standards and integration layers help ensure secure and efficient data exchange among hospital information systems, educational platforms, and digital governance infrastructures.

In practice, however, the maturity of digital technologies in medical education varies substantially across countries. In general, the adoption of digital technologies in medical education governance may evolve through several stages, beginning with basic digital resource management, followed by platform interoperability and data integration, then the application of learning analytics, and ultimately the use of AI-supported predictive and adaptive governance tools (40). In highly developed systems such as the United Kingdom, integrated learning analytics platforms and national education databases have enabled large-scale monitoring of medical training outcomes (16). In contrast, many institutions in developing or transitional systems remain at an earlier stage of digital adoption, where educational platforms function primarily as repositories for teaching resources rather than as integrated data-driven governance tools (41). These differences highlight the importance of technological readiness and infrastructure investment in shaping the effectiveness of digital governance initiatives.

Nevertheless, the feasibility of advanced digital platforms and AI-driven assessment systems may differ substantially across institutional and regional contexts. Institutions with limited digital infrastructure may adopt incremental or hybrid approaches when integrating these technologies into medical education.

### 5.4 Quality layer: data-driven quality assurance and continuous improvement

The quality layer focuses on monitoring and refining governance outcomes through a data-driven, dynamic quality assurance system.

Building upon intelligent assessment mechanisms, digital monitoring enables visualization and real-time tracking of teaching processes. By continuously recording teaching behaviors and learning data, the monitoring system can identify issues, provide timely feedback, and support ongoing quality enhancement (21). This ultimately leads to a closed-loop quality assurance system encompassing the entire medical talent development process, enhancing both the stability and sustainability of the governance structure. To evaluate AI-driven education systems within this quality loop, institutions should define measurable performance and governance indicators. Key dimensions include effectiveness (e.g., improvement in competency progression or remediation timeliness), reliability (agreement between AI outputs and expert ratings), fairness (performance consistency across trainee subgroups and training sites), explainability (availability of interpretable rationales for AI recommendations), and safety governance (human-in-the-loop override and appeal mechanisms) (42, 43). Continuous monitoring for model drift and periodic recalibration are also necessary to ensure sustained validity across cohorts and institutional contexts (44).

By synergizing the institutional, mechanism, technological, and quality layers, this digital governance framework offers a structured and actionable roadmap for enhancing medical-education collaboration. In practice, however, the adoption of such governance models may need to be tailored to local institutional conditions, policy environments, and resource availability. Its core value lies in bridging the disconnect between teaching and clinical practice, reinforcing data-driven decision-making, and providing a robust foundation for the deep integration of smart education technologies.

## 6 Ethical, fairness, and security issues in data governance and intelligent assessment

**FIGURE 4:**
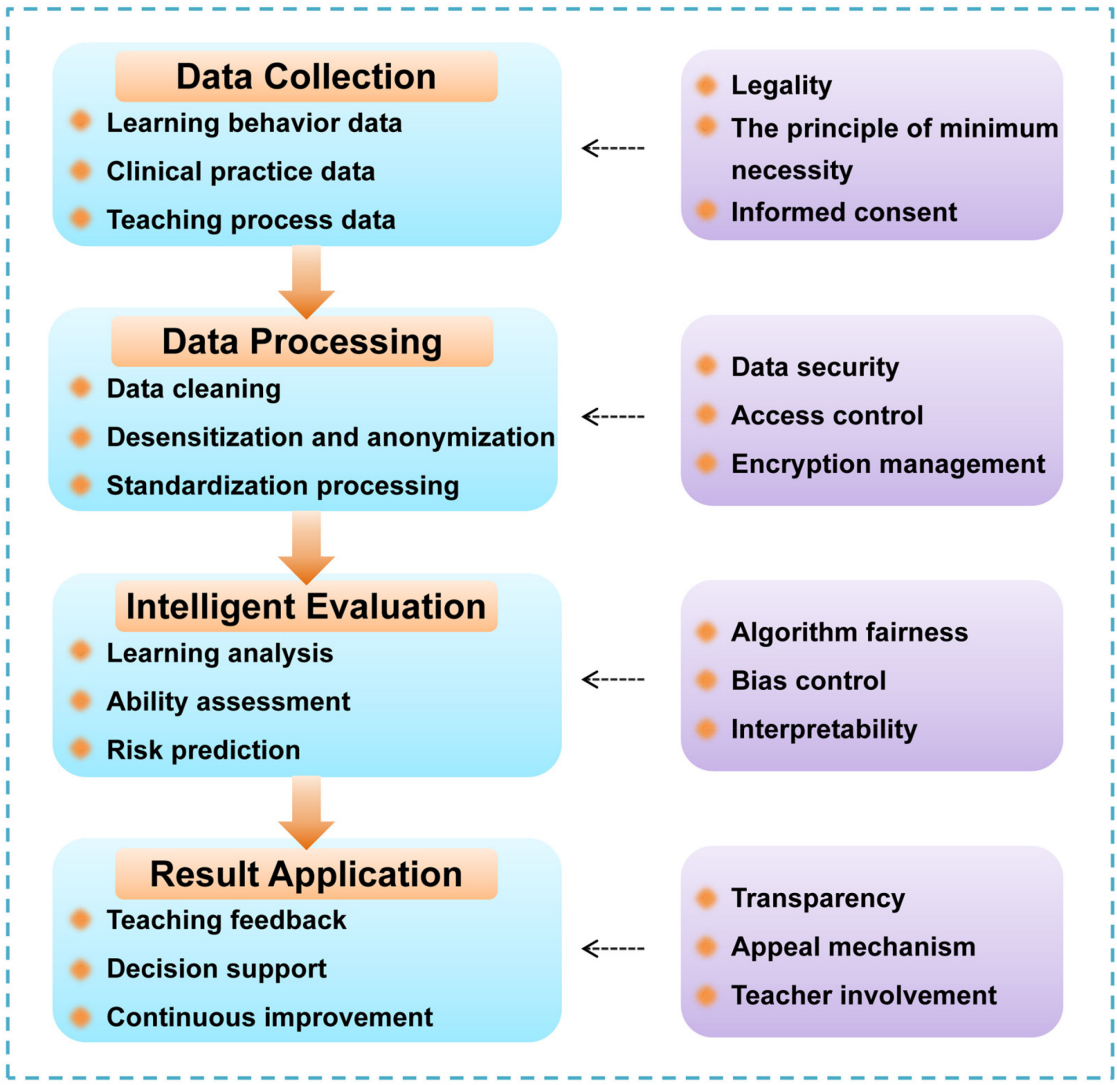
Operational flow of data governance and intelligent assessment.

As digital governance systems continue to evolve in medical education, the expanded use of data and intelligent assessment tools presents a series of ethical and security challenges. With the increasing collection, integration, and analysis of both instructional and clinical data, concerns related to privacy protection, algorithmic fairness, and transparency have become critical. Figure 4 illustrates the key stages in the data lifecycle—from collection to application—where improper design at any stage may compromise the equity and reliability of the governance system.

First, data governance challenges are particularly evident in areas such as privacy risks, lack of standardization, and difficulties in cross-institutional data flow. Medical education data—including learning records, clinical practice logs, and competency assessments—tend to be highly sensitive, multi-sourced, and frequently exchanged (39, 45). Therefore, clear data management protocols must be established to ensure lawful, necessary, and minimal data collection, with transparent use cases and controllable data pathways. Processes such as data cleaning, standardization, and anonymization must be carefully implemented to avoid biases introduced by technical discrepancies or data quality issues. When handling patient-related data, such as medical records and clinical notes, strict access control and encryption mechanisms are essential to meet both compliance and ethical standards. International frameworks, such as the General Data Protection Regulation (GDPR) and the Health Insurance Portability and Accountability Act (HIPAA), offer valuable regulatory references (46, 47).

However, these regulatory frameworks differ in their governance emphasis. The GDPR places strong requirements on data minimization, transparency, and cross-institutional data protection within the European Union, which can influence how educational and clinical data are integrated across institutions (48, 49). In contrast, HIPAA primarily focuses on protecting patient health information within healthcare organizations in the United States (50, 51). These policy differences shape the implementation pathways of digital governance systems, particularly in areas such as data sharing, interoperability standards, and the integration of educational and clinical information platforms.

Despite the potential benefits of digital governance in enhancing coordination and transparency, its implementation may also introduce new institutional tensions (52). For example, the standardization of digital platforms and data governance frameworks may require centralized oversight (53), which may conflict with the traditional autonomy of medical schools and teaching hospitals in areas such as curriculum design and clinical training management (54). Beyond these governance tensions, several practical challenges may also hinder the effective adoption of digital governance systems in medical education (19, 29). Establishing interoperable digital infrastructure often requires substantial financial investment, particularly for institutions with limited technological resources. Variations in faculty digital literacy may also affect the effective use of intelligent educational technologies. Furthermore, institutional resistance to organizational change may slow the integration of data-driven governance systems into existing educational structures. Finally, regulatory complexity and data protection requirements may complicate cross-institutional data sharing and platform interoperability (55). Balancing the need for system-wide coordination with institutional flexibility therefore becomes an important governance challenge.

In addition, while intelligent assessment systems can enhance educational effectiveness, they may also introduce potential risks. Competency-based evaluations supported by learning analytics and AI models provide comprehensive assessments and real-time feedback. However, algorithmic bias and lack of interpretability can undermine fairness. If training datasets lack diversity or if algorithmic logic remains opaque, certain student groups may be systematically misjudged (56, 57). Overreliance on automated results may also marginalize instructors’ roles in judgment and personalized support, weakening the professional and human-centered nature of educational assessment (58). Accordingly, routine algorithmic audits—such as bias testing across cohorts, documentation of model inputs and outputs, and the publication of transparency reports—should be incorporated into institutional governance frameworks to complement educational effectiveness evaluation and ensure accountability in AI-assisted assessment systems.

To further strengthen ethical governance of AI-assisted educational systems, several technical and institutional safeguards should be considered. Explainable artificial intelligence (XAI) approaches can enhance transparency by enabling interpretable model outputs and clearer decision pathways, allowing educators and trainees to better understand how algorithmic recommendations are generated (59). In addition, bias mitigation techniques—such as balanced training datasets, fairness-aware model design, and continuous monitoring of model performance across demographic groups—can help reduce the risk of systematic discrimination (60, 61). At the governance level, institutional audit frameworks may be implemented to periodically evaluate model validity, fairness, and robustness, while clearly defined accountability mechanisms should specify the responsibilities of institutions, system developers, and educational administrators in overseeing AI-assisted decision-making processes (62).

In addition, data-driven governance may generate unintended consequences if not carefully implemented. Excessive reliance on performance metrics and learning analytics may encourage managerial approaches that prioritize quantifiable indicators over complex educational processes (63). Such systems may also raise concerns regarding academic autonomy, perceived surveillance of teaching activities, and potential resistance from faculty members who may question the fairness or transparency of algorithmic assessment systems (54, 64). Addressing these challenges requires governance mechanisms that combine technological innovation with participatory decision-making and transparent evaluation standards.

Furthermore, digital inequality—commonly referred to as the “digital divide”—poses significant equity concerns in the digital learning environment. Variations in technological infrastructure and digital literacy across regions and institutions may lead to disparities in students’ access to resources, clinical opportunities, and assessment formats, thereby affecting the overall fairness of medical talent development (65). To mitigate such risks, intelligent systems must be designed with transparent processes, explainable outputs, and built-in mechanisms for feedback and appeals, enabling both educators and learners to understand and engage with the evaluation process.

In summary, ethical, security, and fairness considerations permeate the entire lifecycle of digital governance and intelligent assessment in medical education. Establishing clear data standards, ensuring algorithmic transparency and justice, strengthening data security measures, and addressing regional disparities through sound policy frameworks are essential to the sustainable and equitable advancement of digital medical education.

## 7 Future pathways for digitally reshaping medical talent cultivation

As governance systems mature, the digital transformation of medical education is evolving beyond the level of technological tools, shifting toward a systemic restructuring centered on learning objectives, instructional models, and collaborative mechanisms. Building upon a well-defined governance framework and standardized data protocols, the transformation may be strategically advanced through the following four pathways. However, the feasibility and pace of implementing these strategies may vary across regions and institutions depending on differences in technological infrastructure, financial resources, and policy environments.

First, instructional models may evolve toward greater precision and openness. Leveraging learning analytics and AI, teaching systems can potentially tailor content and learning trajectories to individual learner profiles, thereby enhancing the precision of personalized support. The broad application of virtual simulation and AR/VR technologies improves the immersion and safety of clinical skill training, while blended learning expands curricular flexibility and interactivity (66, 67). This transition redefines the role of educators from content deliverers to designers of learning experiences, reinforcing adaptability and learner agency.

Second, competency frameworks (4) will shift from knowledge-oriented to capability-driven paradigms. The digital learning environment broadens the scope of essential competencies to include digital literacy, data-informed judgment, and human-machine collaboration. With the support of structured data and intelligent assessment systems, competency evaluation becomes more comprehensive and traceable, allowing the integration of professional knowledge, clinical procedures, communication skills, and ethical reasoning into a unified evaluation framework. This strengthens the alignment between learning outcomes and clinical practice.

Third, collaborative mechanisms between medical education stakeholders may be increasingly integrated through digital platforms. Standardized data interfaces and interoperable platforms enable dynamic cross-institutional coordination of curriculum development, clinical training arrangements, and teaching feedback. Medical schools and teaching hospitals can co-conduct instructional research and curriculum optimization via shared data platforms, while hospitals can use real-world data to contribute to the formative assessment of students, thereby advancing an integrated “teaching–learning–assessment” system.

Fourth, policy and institutional frameworks will provide essential external support and equity safeguards. National-level digital education platforms, unified data standards, and robust quality monitoring systems can collectively enhance the efficiency and quality of educational governance. Simultaneously, initiatives such as faculty digital literacy training and interregional resource-sharing policies can help narrow technological gaps between institutions, promoting fairness and sustainable development in medical education.

Overall, the digital transformation of medical education is not merely a technological upgrade but a fundamental shift in educational philosophy, competency frameworks, and governance models. Through synergistic efforts in instructional innovation, competency restructuring, collaborative integration, and institutional reinforcement, the medical talent cultivation system is poised to evolve toward greater intelligence, precision, and sustainability.

## 8 Limitations

This study has several limitations. First, as a narrative review, the literature selection process may be subject to potential selection bias and does not follow a fully systematic review methodology. Second, the four-layer digital governance framework proposed in this study is conceptual in nature and has not yet been empirically validated through case studies or quantitative analyses. Future research could examine its applicability through empirical investigations in different institutional contexts. Third, the literature discussed in this review primarily reflects experiences from several representative regions, and governance models may vary across countries with different policy environments and levels of digital maturity. Therefore, the conclusions should be interpreted with consideration of regional differences.

## 9 Future research directions

Future research should prioritize empirical validation of the proposed digital governance framework in diverse institutional and regulatory contexts. Comparative studies across countries or regions could help clarify how governance structures, regulatory environments, and technological infrastructures shape the development of digital medical education systems. In addition, quantitative evaluations of learning analytics, intelligent assessment systems, and AI-assisted instructional models are needed to assess their impact on educational outcomes and clinical competency development. Further research should also explore governance strategies that balance technological innovation with ethical considerations, including algorithmic fairness, data privacy, and institutional accountability.

## 10 Conclusion

The advancement of smart education presents a pivotal opportunity for the transformation of medical education. Its core value lies in driving systemic restructuring across instructional organization, competency development, and quality assurance. This paper has reviewed the current governance challenges in medical-education collaboration, highlighting how fragmented structures, unbalanced resource allocation, and persistent data silos constrain the quality of talent cultivation. It underscores the necessity of building a robust digital governance framework.

Focusing on institutional design, collaborative mechanisms, technological platforms, and quality monitoring, the study proposes a four-layer governance structure aimed at effectively bridging teaching and clinical practice, as well as linking data with assessment. Additionally, it systematically addresses critical challenges in data governance and intelligent assessment—particularly those concerning privacy, fairness, and algorithmic transparency—emphasizing the importance of maintaining a dynamic balance between technological innovation and ethical safeguards.

In essence, digital transformation will continue to propel medical education toward greater intelligence, precision, and integration. Only through stable institutional foundations, sound governance structures, and a sustainable data ecosystem can the full potential of smart education be realized—ultimately serving as a driving force for deep, lasting reform in medical talent cultivation.
